# Supplementary material for: Machine Learning Accelerates Crystallization for Structure Determination
Source: Angew Chem Int Ed Engl. 2026 May 4;65(25):e1218503. doi: 10.1002/anie.1218503 (PMC13266958; doi:10.1002/anie.1218503)

checkCIF/PLATON report

Structure factors have been supplied for datablock(s) lcz115

THIS REPORT IS FOR GUIDANCE ONLY. IF USED AS PART OF A REVIEW PROCEDURE FOR PUBLICATION, IT SHOULD NOT REPLACE THE EXPERTISE OF AN EXPERIENCED CRYSTALLOGRAPHIC REFEREE.

No syntax errors found. CIF dictionary Interpreting this report

Datablock: lcz115

Bond precision:	C-C = 0.0326 A		Wavelength=1.54184
Cell:	a=13.0226(1)	b=22.6240(2)	c=48.3112(5)
	alpha=90	beta=90	gamma=90
Temperature:	100 K		

	Calculated	Reported
Volume	14233.6(2)	14233.6(2)
Space group	C 2 2 21	C 2 2 21
Hall group	C 2c 2	C 2c 2
Moiety formula	C110 H81 Ag12 F72 N25 O10 S	2(C15 H3 Ag3 F18 N6), C6 H4.5 N0.5 S0.5, C19 H30 O5
Sum formula	C110 H81 Ag12 F72 N25 O10 S	C55 H40.50 Ag6 F36 N12.50 O5 S0.50
Mr	4607.50	2303.75
Dx,g cm-3	2.150	2.150
Z	4	8
Mu (mm-1)	14.516	14.516
F000	8896.0	8896.0
F000’	8939.37	
h,k,lmax	16,28,61	16,28,60
Nref	15222[8234]	13243
Tmin,Tmax	0.170,0.175	0.384,1.000
Tmin’	0.076	

```
Correction method= # Reported T Limits: Tmin=0.384 Tmax=1.000
AbsCorr = MULTI-SCAN

Data completeness= 1.61/0.87        Theta(max)= 77.702

R(reflections)= 0.0914( 12766)                  wR2(reflections)=
                                                0.2202( 13243)
S = 1.195                   Npar= 976
```

The following ALERTS were generated. Each ALERT has the format
test-name_ALERT_alert-type_alert-level.
Click on the hyperlinks for more details of the test.

Alert level B

```
PLAT342_ALERT_3_B Low Bond Precision on  C-C Bonds ..............    0.03255 Ang.
```

Author Response: Disordered structure.

Alert level C

```
PLAT029_ALERT_3_C _diffrn_measured_fraction_theta_full value Low .      0.975 Why?
PLAT041_ALERT_1_C Calc. and Reported SumFormula    Strings  Differ     Please Check
              Calc: C110 H81 Ag12 F72 N25 O10 S
              Rep.: C55 H40.50 Ag6 F36 N12.50 O5 S0.50
PLAT042_ALERT_1_C Calc. and Reported MoietyFormula Strings  Differ     Please Check
              Calc: C110 H81 Ag12 F72 N25 O10 S
              Rep.: 2(C15 H3 Ag3 F18 N6), C6 H4.5 N0.5 S0.5, C19 H
                    30 O5
PLAT090_ALERT_3_C Poor Data / Parameter Ratio (Zmax > 18) ........       7.90 Note
PLAT213_ALERT_2_C Atom F024            has ADP max/min Ratio .....        3.5 prolat
PLAT220_ALERT_2_C NonSolvent   Resd 1  C   Ueq(max)/Ueq(min) Range        3.6 Ratio
PLAT220_ALERT_2_C NonSolvent   Resd 1  F   Ueq(max)/Ueq(min) Range        4.6 Ratio
PLAT220_ALERT_2_C NonSolvent   Resd 1  N   Ueq(max)/Ueq(min) Range        3.4 Ratio
PLAT230_ALERT_2_C Hirshfeld Test Diff for    F01I     --C01W     .        5.1 s.u.
PLAT234_ALERT_4_C Large Hirshfeld Difference N00H     --C029     .       0.19 Ang.
PLAT234_ALERT_4_C Large Hirshfeld Difference C022     --C026     .       0.23 Ang.
PLAT242_ALERT_2_C Low    'MainMol' Ueq as Compared to Neighbors of       C022 Check
PLAT362_ALERT_2_C Short  C(sp3)-C(sp2) Bond  C01O     - C02X     .       1.36 Ang.
PLAT362_ALERT_2_C Short  C(sp3)-C(sp2) Bond  C01S     - C02L     .       1.39 Ang.
PLAT362_ALERT_2_C Short  C(sp3)-C(sp2) Bond  C029     - C02Q     .       1.40 Ang.
PLAT362_ALERT_2_C Short  C(sp3)-C(sp2) Bond  C01P     - C01M     .       1.41 Ang.
PLAT362_ALERT_2_C Short  C(sp3)-C(sp2) Bond  C01F     - C035     .       1.36 Ang.
PLAT906_ALERT_3_C Large K Value in the Analysis of Variance ......      3.460 Check
PLAT911_ALERT_3_C Missing FCF Refl Between Thmin & STh/L=    0.600        174 Report
                 1  3  0,  15  3  0,   0  4  0,   2  4  0,   1  5  0,   0  6  0,
                 2  6  0,  15  7  0,   0  8  0,  12  8  0,  14  8  0,  13  9  0,
                 0 10  0,  14 12  0,  10 18  0,   9 19  0,  11 19  0,  10 20  0,
                 9 21  0,   0  2  1,   0  4  1,   2  4  1,   0  6  1,   0  8  1,
                12  8  1,  14  8  1,  13  9  1,  10 18  1,   9 19  1,  11 19  1,
                   (  144 More Missing: see the .ckf listing file)
```

```
PLAT915_ALERT_3_C No Flack x Check Done: Low Friedel Pair Coverage        79 %
PLAT971_ALERT_2_C Check Calcd Resid. Dens.  1.36Ang From C026            2.34 eA-3
PLAT971_ALERT_2_C Check Calcd Resid. Dens.  0.78Ang From Ag01            2.25 eA-3
PLAT971_ALERT_2_C Check Calcd Resid. Dens.  0.96Ang From Ag06            1.76 eA-3
PLAT971_ALERT_2_C Check Calcd Resid. Dens.  0.83Ang From Ag04            1.75 eA-3
PLAT971_ALERT_2_C Check Calcd Resid. Dens.  0.98Ang From Ag05            1.74 eA-3
PLAT971_ALERT_2_C Check Calcd Resid. Dens.  1.07Ang From Ag01            1.64 eA-3
PLAT971_ALERT_2_C Check Calcd Resid. Dens.  0.83Ang From Ag04            1.64 eA-3
PLAT971_ALERT_2_C Check Calcd Resid. Dens.  0.98Ang From Ag06            1.57 eA-3
PLAT971_ALERT_2_C Check Calcd Resid. Dens.  1.40Ang From Ag02            1.54 eA-3
PLAT972_ALERT_2_C Check Calcd Resid. Dens.  1.60Ang From Ag01           -1.52 eA-3
PLAT977_ALERT_2_C Check Negative Difference Density on H028      .      -0.35 eA-3
PLAT977_ALERT_2_C Check Negative Difference Density on H01C      .      -0.47 eA-3
PLAT977_ALERT_2_C Check Negative Difference Density on H02C      .      -0.47 eA-3
PLAT977_ALERT_2_C Check Negative Difference Density on H02V      .      -0.39 eA-3
PLAT977_ALERT_2_C Check Negative Difference Density on H02L      .      -0.43 eA-3
```

Alert level G

```
PLAT003_ALERT_2_G Number of Uiso or U(i,j)  Restrained non-H-Atoms         60 Report
PLAT007_ALERT_5_G Number of Unrefined Donor-H Atoms ..............          2 Report
              H1    H00K
PLAT045_ALERT_1_G Calculated and Reported Z Differ by a Factor ...      0.500 Check
PLAT083_ALERT_2_G SHELXL Second Parameter in WGHT  Unusually Large    1392.47 Why ?
PLAT142_ALERT_4_G s.u. on b - Axis Small or Missing ..............    0.00020 Ang.
PLAT177_ALERT_4_G The CIF-Embedded .res File Contains DELU Records          3 Report
PLAT186_ALERT_4_G The CIF-Embedded .res File Contains ISOR Records          9 Report
PLAT192_ALERT_3_G A Non-default DELU Restraint Value for SecondPar     0.0200 Report
PLAT192_ALERT_3_G A Non-default DELU Restraint Value for SecondPar     0.0200 Report
PLAT192_ALERT_3_G A Non-default DELU Restraint Value for SecondPar     0.0200 Report
PLAT242_ALERT_2_G Low    'MainMol' Ueq as Compared to Neighbors of       C01M Check
PLAT242_ALERT_2_G Low    'MainMol' Ueq as Compared to Neighbors of       C01W Check
PLAT242_ALERT_2_G Low    'MainMol' Ueq as Compared to Neighbors of       C01Y Check
PLAT242_ALERT_2_G Low    'MainMol' Ueq as Compared to Neighbors of       C026 Check
PLAT242_ALERT_2_G Low    'MainMol' Ueq as Compared to Neighbors of       C027 Check
PLAT242_ALERT_2_G Low    'MainMol' Ueq as Compared to Neighbors of       C02G Check
PLAT242_ALERT_2_G Low    'MainMol' Ueq as Compared to Neighbors of       C02P Check
PLAT242_ALERT_2_G Low    'MainMol' Ueq as Compared to Neighbors of       C02Q Check
PLAT242_ALERT_2_G Low    'MainMol' Ueq as Compared to Neighbors of       C02X Check
PLAT242_ALERT_2_G Low    'MainMol' Ueq as Compared to Neighbors of       C035 Check
PLAT333_ALERT_2_G Large Aver C6-Ring C-C Dist C01Q     -C02K     .       1.43 Ang.
PLAT335_ALERT_2_G Check Large C6 Ring C-C Range C01Q     -C02K           0.20 Ang.
PLAT343_ALERT_2_G Unusual sp3      Angle Range in Main Residue for       C026 Check
PLAT434_ALERT_2_G Short Inter HL..HL Contact F00A     ..F00A     .       2.80 Ang.
                                               x,1-y,1-z  =      4_566 Check
PLAT434_ALERT_2_G Short Inter HL..HL Contact F00B     ..F02U     .       2.78 Ang.
                                          1/2+x,-1/2+y,z  =      5_545 Check
PLAT434_ALERT_2_G Short Inter HL..HL Contact F019     ..F02M     .       2.76 Ang.
                                          1/2+x,-1/2+y,z  =      5_545 Check
PLAT720_ALERT_4_G Number of Unusual/Non-Standard Labels ..........        154 Note
              Ag01    Ag02    Ag03    Ag04    Ag05    Ag06    S007    F008
              F009    F00A    F00B    F00E    F00F    F00G    F00I    N00J
              C01O    C028    H028    C022    N01Z    O00K    H00K    F00L
              F00M    F00N    F00O    O00P    F00Q    F00R    N00T    N00X
              C01K    C02C    H02Q    C02B    N00U    C01N    C01H    H01H
              C01S    N00S    F00V    O00W    F00Z    O010    F011    F012
              F013    F015    O016    F019    F01A    F01B    C01C    H01C
```

```
              C029    N00H    N00C    C01J    F01D    F01E    C01G    N00Y
              N00D    C01P    C025    H025    F01I    C01L    C02D    H02R
              C021    H021    C01T    H01T    C02R    H02S    C017    C01M
              C01Q    C01R    H01A    H01B    C01U    F01V    C01W    C01X
              C01Y    C020    H02A    H02B    F023    F024    C026    C027
              C02A    H02C    H02D    H02E    C02E    H02F    H02G    F02F
              C02G    C02I    F02J    C02K    C02L    F02M    F02N    C02O
              H02H    H02I    C02P    C02Q    C02S    H02J    H02K    C02T
              F02U    C02V    H02V    C01F    N018    N014    C02Z    C02W
              H02L    H02M    C02X    C02Y    H02N    H02O    H02P    C031
              H03A    H03B    H03C    C033    H03D    H03E    F034    C035
              C036    H03F    H03G    C037    C038    H03H    H03I    C03A
              H03J    H03K
PLAT860_ALERT_3_G Number of Least-Squares Restraints .............        357 Note
PLAT910_ALERT_3_G Missing FCF Reflection(s) Below Theta(Min)[Deg]=       3.92 Note
                0  2  0,   0  0  2,   0  0  4,
PLAT912_ALERT_4_G Missing # of FCF Reflections Above STh/L=  0.600        340 Note
PLAT930_ALERT_2_G FCF-based   Twin Law ( 1 1 0)         Est.d BASF       0.06 Check
PLAT930_ALERT_2_G FCF-based   Twin Law          [ 1 1 0] Est.d BASF       0.07 Check
PLAT930_ALERT_2_G FCF-based   Twin Law          [ 3 1 0] Est.d BASF       0.06 Check
PLAT930_ALERT_2_G FCF-based   Twin Law          [ 3 1 0] Est.d BASF       0.06 Check
PLAT931_ALERT_5_G CIFcalcFCF  Twin Law ( 1 1 0)         Est.d BASF       0.06 Check
PLAT931_ALERT_5_G CIFcalcFCF  Twin Law          [ 1 1 0] Est.d BASF       0.07 Check
PLAT931_ALERT_5_G CIFcalcFCF  Twin Law          [ 3 1 0] Est.d BASF       0.06 Check
PLAT931_ALERT_5_G CIFcalcFCF  Twin Law          [ 3 1 0] Est.d BASF       0.06 Check
PLAT933_ALERT_2_G Number of HKL-OMIT Records in Embedded .res File          1 Note
                2  6  0,
PLAT941_ALERT_3_G Average HKL Measurement Multiplicity ..........         3.0 Low
PLAT969_ALERT_5_G The 'Henn et al.' R-Factor-gap value ..........      4.059 Note
              Predicted wR2: Based on SigI**2  5.43 or SHELX Weight 18.43
PLAT978_ALERT_2_G Number C-C Bonds with Positive Residual Density.          0 Info
```

```
   0 ALERT level A = Most likely a serious problem - resolve or explain
   1 ALERT level B = A potentially serious problem, consider carefully
  35 ALERT level C = Check. Ensure it is not caused by an omission or oversight
  42 ALERT level G = General information/check it is not something unexpected

   3 ALERT type 1 CIF construction/syntax error, inconsistent or missing data
  50 ALERT type 2 Indicator that the structure model may be wrong or deficient
  12 ALERT type 3 Indicator that the structure quality may be low
   7 ALERT type 4 Improvement, methodology, query or suggestion
   6 ALERT type 5 Informative message, check
```

Validation response form

Please find below a validation response form (VRF) that can be filled in and pasted into your CIF.

```
# start Validation Reply Form
_vrf_PLAT029_lcz115
;
PROBLEM: _diffrn_measured_fraction_theta_full value Low .      0.975 Why?
RESPONSE: ...
;
_vrf_PLAT041_lcz115
;
```

```
PROBLEM: Calc. and Reported SumFormula    Strings  Differ     Please Check
RESPONSE: ...
;
_vrf_PLAT042_lcz115
;
PROBLEM: Calc. and Reported MoietyFormula Strings  Differ     Please Check
RESPONSE: ...
;
_vrf_PLAT090_lcz115
;
PROBLEM: Poor Data / Parameter Ratio (Zmax > 18) ........       7.90 Note
RESPONSE: ...
;
_vrf_PLAT213_lcz115
;
PROBLEM: Atom F024            has ADP max/min Ratio .....        3.5 prolat
RESPONSE: ...
;
_vrf_PLAT220_lcz115
;
PROBLEM: NonSolvent   Resd 1  C   Ueq(max)/Ueq(min) Range        3.6 Ratio
RESPONSE: ...
;
_vrf_PLAT230_lcz115
;
PROBLEM: Hirshfeld Test Diff for    F01I     --C01W     .        5.1 s.u.
RESPONSE: ...
;
_vrf_PLAT234_lcz115
;
PROBLEM: Large Hirshfeld Difference N00H     --C029     .       0.19 Ang.
RESPONSE: ...
;
_vrf_PLAT242_lcz115
;
PROBLEM: Low    'MainMol' Ueq as Compared to Neighbors of       C022 Check
RESPONSE: ...
;
_vrf_PLAT362_lcz115
;
PROBLEM: Short  C(sp3)-C(sp2) Bond  C01O     - C02X     .       1.36 Ang.
RESPONSE: ...
;
_vrf_PLAT906_lcz115
;
PROBLEM: Large K Value in the Analysis of Variance ......      3.460 Check
RESPONSE: ...
;
_vrf_PLAT911_lcz115
;
PROBLEM: Missing FCF Refl Between Thmin & STh/L=    0.600        174 Report
RESPONSE: ...
;
_vrf_PLAT915_lcz115
;
PROBLEM: No Flack x Check Done: Low Friedel Pair Coverage         79 %
RESPONSE: ...
```

```
;
_vrf_PLAT971_lcz115
;
PROBLEM: Check Calcd Resid. Dens.  1.36Ang From C026           2.34 eA-3
RESPONSE: ...
;
_vrf_PLAT972_lcz115
;
PROBLEM: Check Calcd Resid. Dens.  1.60Ang From Ag01          -1.52 eA-3
RESPONSE: ...
;
_vrf_PLAT977_lcz115
;
PROBLEM: Check Negative Difference Density on H028      .      -0.35 eA-3
RESPONSE: ...
;
# end Validation Reply Form
```

It is advisable to attempt to resolve as many as possible of the alerts in all categories. Often the minor alerts point to easily fixed oversights, errors and omissions in your CIF or refinement strategy, so attention to these fine details can be worthwhile. It is up to the individual to critically assess their own results and, if necessary, seek expert advice.

PLATON version of 17/09/2025; check.def file version of 17/09/2025

---

duplicate check

No duplication found

Datablock lcz115 - ellipsoid plot

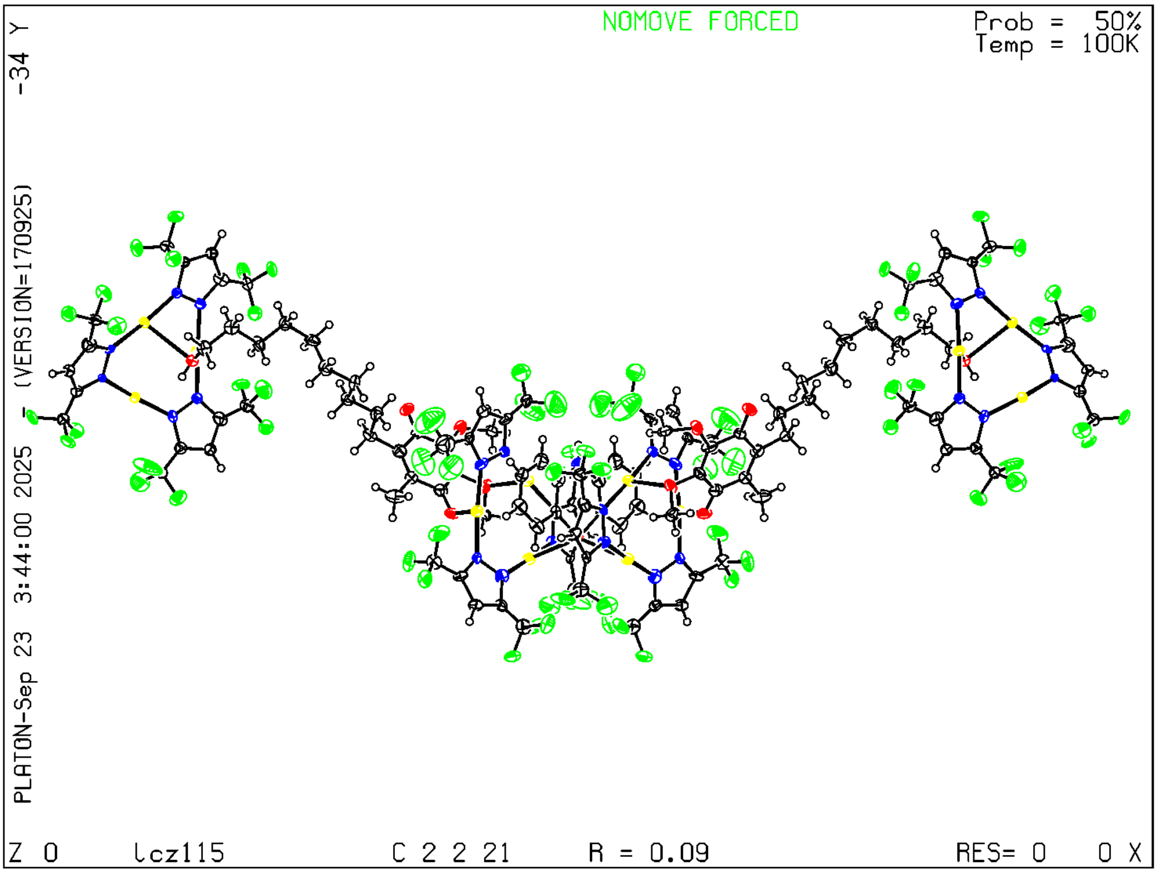

Supplement: Supplementary file 2 — Supporting File 2: anie72462‐sup‐0002‐DataFile.zip. [file ANIE-65-e1218503-s002.zip › supplementary_materials_crystallographic_data submit.zip!/cif_and_checkcif(1-114)/Ag3Pz3_87_103/Ag3Pz3_87_103_checkcif.pdf]
